# The Platformized Commercial Persona: CEO Visibility, Fan Labor, and Consumer Engagement in Short-Video Social Commerce

**DOI:** 10.3390/bs16091607

**Published:** 2026-09-09

**Authors:** Hongbo Liao, Jiangmin Ding

**Affiliations:** 1 School of Journalism, Communication University of China, Beijing 100024, China; 202610050301011@mails.cuc.edu.cn; 2 School of Business, Pusan National University, Busan 46241, Republic of Korea

**Keywords:** social commerce, CEO influencerization, platformized commercial persona, fan labor, consumer engagement

## Abstract

Research on social commerce, influencer marketing, consumer–brand engagement, and CEO social media communication has rapidly expanded. However, less is known regarding how entrepreneurial leaders’ personal short-video accounts operate as platformized interfaces that organize consumer interaction. This study examines how CEO short-video communication produces an observable, collectively sustained commercial persona and connects executive visibility with fan labor, consumer–brand engagement, and consumption imagination. Using the Douyin account of a leading Chinese technology entrepreneur as a case, this study analyzes 372 short videos posted up to 15 August 2024, and 185,561 valid first-level comments. It combines structured content analysis, intercoder reliability testing, BERTopic-assisted topic location, manual thematic validation, and text-based emotional intensity analysis. Work footage, scenes of everyday life, casual presentations, and product props translate entrepreneurial authority into an approachable, platformized commercial persona. In comment sections, users perform informational, relational, affective, memetic, mobilizing, and defensive labor through product requests, familiar address, emotional identification, memes, consumption mobilization, and brand defense. These practices stabilize CEO personas, circulate product meanings, and create consumption imagination, understood as public projections of saving for, ordering, using, or affiliating with products rather than evidence of actual purchases. Emotional identification, consumption mobilization, and brand defense display higher observable emotional intensity than product requests. This study contributes a case-derived conceptual framework demonstrating how CEO identity capital, platform affordances, fan labor, and product-centered imagination are assembled in short-video social commerce.

## 1. Introduction

The rise in short-video platforms has changed how organizations, leaders, and consumers connect. In traditional corporate communication, corporate information is typically distributed through formal channels, such as press releases, public relations campaigns, corporate advertising, and executive interviews. Short-video platforms reorganize this relationship by placing entrepreneurial leaders in a public environment shaped by algorithmic recommendations, quantified interactions, affective expression, and everyday narratives. Recommendation feeds and engagement buttons are not neutral conduits. They make some organizational actors more visible, provide lightweight response mechanisms, and organize repeated encounters between platform users and public figures (Scharlach & Hallinan, 2023; Schellewald, 2023).

This transformation is particularly visible in China, where short-video platforms have become major infrastructure for everyday media consumption. According to the 55th Statistical Report on China’s Internet Development, China had 1.04 billion short-video users by December 2024, accounting for 93.8% of all Internet users (China Internet Network Information Center, 2025). In this platform ecology, entrepreneurs such as technology founders, service-platform executives, and education-business leaders enter users’ daily media flows through short videos, livestreams, and comment-section interactions. Entrepreneurial discourse is no longer confined to corporate announcements or financial news. It is translated into everyday questions, such as what the CEO is wearing, where the CEO is travelling, whether a product can be cheaper, and when a product will be delivered.

Although studies have examined several relevant strands, they have often been developed separately. Social-commerce research commonly foregrounds transaction-oriented engagement (Alghizzawi et al., 2024; Shen, 2023), platform-enabled shopping processes (Dong et al., 2024), and purchase-related outcomes (Tran & Uehara, 2023; He & Jin, 2024; Luo et al., 2025). Influencer-marketing research generally emphasizes ordinary creators (Ki & Kim, 2019; Sokolova & Kefi, 2020; Leung et al., 2022), lifestyle credibility (Lou & Yuan, 2019), branded persuasion (Lou & Yuan, 2019), and endorsement effectiveness (Sokolova & Kefi, 2020). Consumer–brand engagement studies conceptualize commenting, sharing, affective participation, and community activity as relationship-building and value co-creation (Brodie et al., 2013; Hollebeek et al., 2014). CEO social media research typically approaches executive visibility through organization–public relationships, reputation, leadership communication, or social media fame (Men & Tsai, 2016; Yue et al., 2019; Mooney et al., 2026; Lee et al., 2026). Platform-affordance research explains how visibility (Bucher, 2012; Treem & Leonardi, 2013; Duffy & Meisner, 2023), persistence (Treem & Leonardi, 2013), association (Boyd, 2010; Treem & Leonardi, 2013; Scharlach & Hallinan, 2023), recommendation (Schellewald, 2023), and algorithmic sorting organize participation (Van Dijck & Poell, 2013; Duffy & Meisner, 2023; Schellewald, 2023). How these processes converge when an entrepreneurial leader’s personal account becomes a social-commerce interface linking executive visibility, product discussion, fan interaction, and consumption imagination remains unclear. In this setting, engagement is not limited to transactions but includes comments, affective participation, recommendations, identity work, and public defense of products or brands. Therefore, this study explores how an entrepreneur’s short-video presence becomes a digital-commerce resource through which users jointly produce attention, trust, symbolic value, and purchase-oriented imagination.

This study empirically examines the Douyin account of the founder and CEO of a major Chinese technology company. This case is analytically valuable for three reasons. First, it combines organizational authority, founder identity, technological entrepreneurship, and public recognizability. Second, it contains a wide range of videos, including work scenes, factory visits, daily life, travel, school memories, product demonstrations, and user responses. Third, a major product launch created an observable moment in which CEO content, product discussions, comment participation, and market attention became tightly interwoven. Brand and entrepreneur names are discussed in the empirical section only when necessary for case transparency. This study’s analytical focus is on the broader mechanism of CEO short-video communication in social commerce.

Guided by interaction ritual chain theory, platform affordance research, consumer–brand engagement, influencer marketing, and parasocial interaction, the study asks three research questions. RQ1: What content features and interaction resources characterize CEO short videos on a social-commerce platform? RQ2: How do comment-section interactions transform CEO personhood, product issues, and fan/consumer identity into social-commerce engagement rituals? RQ3: How do comment themes vary in observable emotional intensity, and how does this variation help explain consumption imagination and consumer–brand engagement? In this study, persona is conceptualized as a public, mediated, performative, and collectively sustained identity rather than as an individual personality trait or a purely self-authored brand image (Goffman, 1959; Moore et al., 2017). Moving beyond individual entrepreneurs, this study examines how platform visibility, entrepreneurial identity capital, fan participation, and product-centered imagination converge in the construction of a platformized commercial persona in social commerce.

## 2. Literature Review and Research Framework

### 2.1. From Interaction Ritual Chains to Platformized Interaction Rituals

Interaction ritual chain theory originated in Durkheim’s (1912/1995) analysis of ritual and collective emotion and was systematically developed by Collins (2004) to explain how micro-interactions generate emotional energy, solidarity, moral boundaries, and symbolic objects. According to Collins, successful interaction rituals require bodily co-presence, barriers to outsiders, mutual focus of attention, and shared emotional experience. When rituals succeed, participants acquire emotional energy and attach it to symbols that motivate subsequent actions.

Digital platforms modify these conditions. Bodily co-presence is no longer the only basis for ritual participation. Comment sections, livestream rooms, hashtags, and recommendation feeds can create a form of virtual co-presence. Boundaries are produced through physical separation as well as algorithmic sorting, platform rules, fan vocabularies, and interest-based communities. Social media affordances provide not only technical buttons but also perceived visibility, responsiveness, editability, persistence, and association (Sundar, 2008; Boyd, 2010; Treem & Leonardi, 2013; Bucher & Helmond, 2018). Therefore, short-video rituals go beyond offline rituals transferred online to become affective and attentional processes organized by platform mechanisms.

In entrepreneurial short-video communication, interaction rituals are commercially oriented. Entrepreneurs simultaneously act as organizational leaders, product symbols, and public figures. Their videos convey corporate information, personal images, brand value, and consumption cues. Therefore, comment-section emotional energy may contribute not only to fan solidarity but also to brand identification, product attention, and consumption imagination. This study defines platformized interaction rituals as interaction processes based on platform visibility and engagement affordances, focused on entrepreneurial personhood and product issues, driven by the affective participation of fans and consumers, and connected to the production of brand and consumption meanings.

### 2.2. Entrepreneurial Influencers and the Platformized Commercial Persona in Digital Commerce

Research on the social media communication of CEOs and entrepreneurs suggests that direct executive expression can strengthen perceived authenticity, accessibility, and organization–public relationship quality (Men & Tsai, 2016; Yue et al., 2019). Personalized corporate communication and a conversational CEO social media style can also increase perceived approachability and interaction value (Denner & Schneider, 2023; Lee et al., 2026). Unlike traditional corporate public relations statements, an entrepreneur’s personal account often uses first-person narration, everyday settings, and informal language to reduce the distance between the organization and the public. Social media may further turn CEOs into online celebrities when posting activity, affective tone, content diversity, and audience responses create sustained public attention (Mooney et al., 2026). Persona scholarship is useful in this context because it treats public identity as mediated and performative, as well as collective and value-bearing. An online persona is produced not only by the focal actor but also through media forms, audiences, and networked circulation (Goffman, 1959; Moore et al., 2017).

Entrepreneurial influencers differ from ordinary social media influencers. Entrepreneurs have organizational authority, resource visibility, professional responsibility, and public credibility, while simultaneously learning platform grammar, short-video aesthetics, affective address, and fan interaction. The result is not merely an entrepreneurial image or commercial spokesperson. In this study, a platformized commercial persona is defined as a publicly performed and collectively stabilized entrepreneurial identity that is made commercially meaningful through four linked dimensions: organizational authority, platform-shaped approachability, audience co-production, and product-oriented projection. It is organizationally authoritative because the entrepreneur directly carries decision-making power, product responsibility, and corporate reputation; platform-relational because short videos, recommendations, and interaction affordances render the executive visible and addressable; collectively co-produced because comments, memes, requests, and defenses contribute to the persona’s meaning; and commercially projective because attention to the person is repeatedly connected to product use, ownership, and brand participation.

Therefore, the concept is related to, but analytically distinct from, personal branding, CEO celebrity, influencer marketing, parasocial interaction, and consumer–brand engagement. Personal branding emphasizes strategic self-positioning and reputation management (Khamis et al., 2017), whereas the present concept includes algorithmic selection, short-video formats, engagement affordances, and distributed audience labor. CEO celebrity explains the accumulation of attention and status (Mooney et al., 2026), whereas the platformized commercial persona traces how such visibility translates into product feedback, brand participation, and consumption imagination. Influencer marketing explains how creator credibility and branded persuasion affect audiences (Hudders et al., 2021; Leung et al., 2022). However, entrepreneurial influencers directly embody organizational authority and product accountability rather than acting mainly as external endorsers. Although parasocial interaction explains perceived relational closeness (Horton & Wohl, 1956; Labrecque, 2014), the present concept incorporates fan-to-fan interaction, platform architecture, product issues, and collective boundary work. Consumer–brand engagement captures users’ cognitive, emotional, and behavioral investments (Brodie et al., 2013; Hollebeek et al., 2014), whereas platformized commercial personas identify the mediated interface and co-production process through which such engagement is organized. Table 1 summarizes these boundaries.

### 2.3. Fan Labor and Consumption Imagination as Mediating Processes

In this study, fan labor refers to voluntary and usually uncompensated communicative, affective, interpretive, and promotional activities that produce meaning, visibility, sociality, feedback, and economic value around a public figure or brand (Terranova, 2000; Stanfill & Condis, 2014). In comment sections, labor does not require formal employment or explicit self-identification as a fan. It includes informational labor when users ask product questions or propose improvements; relational labor when they sustain familiarity through greetings and personalized address; affective and narrative labor when they circulate inspirational interpretations; cultural labor when they reproduce memes and insider references; promotional labor when they encourage saving, ordering, or recommending; and boundary labor when they defend the brand against criticism. This term is used analytically to identify productive audience contributions without assuming that all participants share the same motives or experience their activity as exploitation.

Consumption imagination is defined as platform-visible discourse in which users project themselves or others into a possible future of acquiring, waiting for, saving for, using, recommending, or publicly affiliating with a product. This concept is related to research on consumption vision and consumption imagery, which examines the mental simulation of product-related behavior and future use (Phillips et al., 1995; Schlosser, 2003; Petrova & Cialdini, 2005). In this study, the term is used more narrowly and interactionally to identify projections that are publicly articulated and socially circulated in comments rather than private mental images. These projections can be reinforced by other users and remain pre-behavioral communicative indicators rather than measures of purchase intention, conversion, or actual consumption.

Fan labor and consumption imagination connect platform interactions to social-commerce outcomes. Platform visibility and engagement affordances make CEO content repeatedly encounterable and readily answerable. Interaction rituals organize shared attention, emotional resonance, group symbols, and boundaries. Recurrent comment labor stabilizes an approachable but authoritative CEO persona, circulates product meanings, and supplies scripts through which users can imagine future acquisition or use. The proposed relationship is interpretive rather than deterministic. Fan labor may enable or intensify consumption imagination, but neither comments nor emotional expression demonstrate purchase behaviors.

### 2.4. Consumer–Brand Engagement, Parasocial Interaction, and Social Commerce

Social media has transformed consumer–brand relationships. Consumer–brand engagement research shows that following, commenting, sharing, and affective expression are not only communication feedback but also forms of relationship building and value co-creation (Brodie et al., 2013; Hollebeek et al., 2014; Leung et al., 2022). More broadly, consumer behavior research suggests that consumption-related responses may develop through interconnected cognitive, affective, and conative processes (Ding & Lee, 2024). Influencer-marketing research indicates that authenticity, credibility, message value, content fit, and parasocial interaction can affect brand attitudes and purchase intentions (De Veirman et al., 2017; Ki & Kim, 2019; Lou & Yuan, 2019; Sokolova & Kefi, 2020). Similarly, short-video marketing research indicates that storyness, entertainment, information relevance, social sharing, and trust influence likes, comments, shares, and purchase-related responses (Boyd, 2010; Dong et al., 2024; Luo et al., 2025). These studies explain important audience outcomes; however, they do not fully clarify how a CEO-centered persona is jointly produced through platform infrastructure and fan/consumer discourse.

Entrepreneurial short videos differ from ordinary influencer commerce. Influencers typically commercialize a lifestyle persona through brand partnerships, whereas entrepreneurs directly represent organizational strategy, product information, and commercial credibility. Their accounts operate simultaneously as public-facing corporate communication, daily fan management, product-feedback channels, and social-commerce activation. This hybridity makes the case useful for theorizing how CEO communication, influencer-like performance, audience labor, and consumer–brand engagement intersect in short-video environments.

Therefore, the framework proceeds in stages rather than treating social-commerce outcomes as a single undifferentiated result. First, platform mechanisms provide visibility, quantification, recommendation opportunities, and short-video content grammar. Second, these conditions enable repeated encounters and support interaction rituals surrounding CEO personhood, product issues, and fan/consumer identity. Third, audience contributions perform informational, relational, affective, memetic, promotional, and defensive labor, thereby stabilizing platformized commercial personas and circulating product meanings. Fourth, consumption imagination becomes visible when users publicly project future purchases, ownership, use, waiting, saving, or recommendations. Consumer–brand engagement intensifies when such projections are connected to emotional identification, community symbols, and boundary maintenance. These are interpretive associations and possible social-commerce outcomes, not direct sales effects or a claim that every exposed user follows the same pathway.

## 3. Materials and Methods

### 3.1. Case Selection, Data Sources, and Sampling Logic

This study examined the Douyin account of Lei Jun, the founder and CEO of Xiaomi, as a case of CEO short-video communication in social commerce. This case was selected because Lei Jun combines founder identity, executive authority, technological entrepreneurship, and public recognizability, while the Xiaomi SU7 launch created a visible connection between CEO content, product discussion, and consumption-oriented user interaction. Therefore, this case is suitable for examining how entrepreneurial identity capital is platformized and converted into consumer engagement resources. The case name is retained in the methods and findings for empirical transparency. The title and theoretical framing are not dependent on the brand name.

The empirical material comprises two main datasets and one supplementary contextual dataset. The first main dataset includes all 372 short videos posted by Lei Jun’s Douyin account up to 15 August 2024. The theme, setting, actors, props, audiovisual elements, duration, likes, comments, and hashtags are recorded for each video. The second main dataset includes first-level comments under these videos. Up to 500 first-level comments were collected from each video using a consistent collection window and the same public-access procedure to avoid overrepresenting a small number of videos with high comment counts while retaining cross-video coverage. The ceiling of 500 was a balancing rule rather than a statistical power threshold. It ensured that all videos were represented, prevented a few viral posts from dominating the corpus, provided near-complete coverage for videos with fewer than 500 accessible first-level comments, and produced a sufficiently large corpus for topic location while remaining feasible for repeated cleaning and manual validation. Across 372 videos, the theoretical ceiling was 186,000 comments. After cleaning, deduplicating, and removing advertising-style texts, meaningless emoji strings, and comments with unclear semantic content, the final dataset comprised 185,561 valid comments. Therefore, the sample should be understood as a capped, platform-visible cross-video corpus rather than a probability sample of all comments.

Comments are defined as platform-visible interaction texts, rather than a random sample of all comments. This distinction is important because popularity ranking, posting time, account status, moderation, and personalized recommendations may influence platform comment representation. Therefore, the analysis does not infer individual-level attitudes or psychological states from comments. It examines observable interaction patterns made visible in the comment environment. Usernames, profile links, account identifiers, and personally identifiable details are not retained in the analytical dataset. The reported representative expressions are paraphrased to prevent reverse identification.

The supplementary contextual dataset comprises publicly circulated third-party regional sales ranking information for the Xiaomi SU7 in April 2024. This dataset is not used to answer the main research questions and not treated as evidence of communication effects. It is reported only in the supplementary contextual section to clarify the boundary between visible platform and market attention (Appendix A).

### 3.2. Content Analysis, Coding Procedure, and Reliability

The short-video analysis used each video as the coding unit. A structured coding sheet was designed based on short-video content analysis, entrepreneurial media image research, platform affordance research, and interaction ritual theory. The coding categories included narrative attributes, presentation status, scene settings, symbolic elements, and communication elements. The categories recorded visible video content and cues that may invite user interaction.

The coding process followed four steps. First, the research team produced an initial codebook containing definitions, inclusion and exclusion criteria, and examples for each category. Second, approximately 20% of the videos (*n* = 74) were randomly selected for precoding to clarify ambiguous distinctions, such as work footage versus business updates, everyday life versus life history, and product display versus product-as-prop. Third, two trained coders independently coded all 372 videos according to the revised coding manual. Fourth, disagreements were discussed item-by-item and adjudicated by a third researcher based on the coding manual and video context (Table 2).

Intercoder reliability was calculated using Cohen’s kappa for nominal variables. The reliability values were as follows: main theme, kappa = 0.86; scene setting, kappa = 0.83; presentation status, kappa = 0.81; symbolic elements, kappa = 0.79; and communication elements, kappa = 0.88. The average value was kappa = 0.83, indicating an acceptable level of agreement for content analysis (Cohen, 1960; Krippendorff, 2019). The coding manual, independent coding records, disagreement log, and adjudication notes were retained to support methodological verification. The anonymized codebook, topic keywords, and validation procedure can be made available upon reasonable request (Table 3).

### 3.3. Topic Modeling, Manual Validation, and Emotional Intensity

Comment analysis followed a computationally assisted and manually validated thematic procedure. Python 3.12 was used to clean 185,561 comments by removing duplicates, advertising-style text, meaningless emoji strings, and very short comments with unclear meanings. Chinese word segmentation, stop-word filtering, synonym merging, and platform slang correction were performed. BERTopic was used as a topic-location tool rather than an automated interpretation system. Comment embeddings were generated using multilingual sentence-transformer paraphrase-multilingual-MiniLM-L12-v2. UMAP was used for dimensionality reduction (n_neighbors = 15, n_components = 5, min_dist = 0.0, metric = cosine, random_state = 42), followed by HDBSCAN density clustering (min_cluster_size = 120, min_samples = 10, cluster_selection_method = eom). Class-based TF-IDF was used to generate topic keywords and representative comments (McInnes et al., 2017; McInnes et al., 2018; Reimers & Gurevych, 2019; Grootendorst, 2022).

BERTopic was used to locate semantically dense comment clusters and support systematic sampling for manual interpretation. Topics were not preset. They were generated using HDBSCAN according to semantic density and then manually reviewed according to four criteria: semantic coherence of keywords, interaction function of representative comments, relevance to entrepreneurial personhood/product issues/fan identity, and sufficient scale and theoretical interpretability. Noise clusters consisting primarily of lottery comments, reposts, meaningless jokes, pure emoji strings, or platform noise were excluded. Semantically similar clusters were merged following manual validation (Table 4).

For each candidate topic, 500 comments were randomly sampled by topic probability strata, and an additional 100 high-like and 100 high-reply comments were reviewed to restore interaction context. Two researchers independently assigned candidate topics to functional themes, and disagreements were resolved through discussion. The final comment themes were product requests, familiar address, emotional identification, memetic interactions, consumption mobilization, and brand defense. A dominant-theme rule was applied, implying that each comment was counted in only one primary category. This rule avoids inflating frequencies when one comment contained product information and affective expression.

An exploratory emotional intensity indicator was added to operationalize the emotional energy implied by interaction ritual chain theory. Emotional intensity does not measure users’ psychological states but captures observable affective expression in text. A public Chinese sentiment lexicon provides initial positive-emotion and intensification vocabularies (Tausczik & Pennebaker, 2010). During manual topic validation, the two researchers compiled platform-specific expressions that recurred across multiple videos or themes and had a stable interactional function after contextual inspection, including action expressions such as “rush,” “place an order,” and “save money,” and boundary expressions such as “do not smear” and “serious product-making.” Ambiguous slang, irony, and expressions whose functions changed across contexts were retained only for manual interpretation and not treated as automatic dictionary hits. Four components were calculated for each comment c: positive affect (Pos_c), lexical intensification through degree adverbs or high-intensity emotion words (Int_c), punctuation/emoji reinforcement (PuncEmoji_c), and action-or-boundary orientation (Orient_c). The components were measured on different raw scales because each was standardized before combination. The composite had an equal-weight arithmetic mean, EI_c = [z(Pos_c) + z(Int_c) + z(PuncEmoji_c) + z(Orient_c)]/4. Equal weighting was used because no validated theoretical basis justified privileging one textual component over another, and the index was intended as an auxiliary comparative device rather than a psychometric scale. Theme-level interpretation relied on both the composite pattern and separate component ratios reported in Table 5; therefore, no conclusion depended on the composite score alone.

## 4. Results

### 4.1. From Entrepreneurial Authority to an Everyday Organizational Persona

Content analysis shows that Lei Jun’s short videos on Douyin are not dominated by formal corporate announcements or grand business narratives. Instead, they emphasize work processes, everyday scenes, and approachable expression. Work footage accounted for 47.53% of the videos, and everyday life accounted for 32.67%, comprising more than 80% of the sample. Business updates and life-history content accounted for 2.97% each. These results suggest that the account’s communication strategy is not to continuously issue corporate information but to transform work scenes, travel, factory visits, product use, and everyday details into content that is watchable, commentable, and affectively participatory.

The presentation of the entrepreneur also tends toward approachability. Casual clothing appeared in 70.30% of the videos, and facial/expressive cues coded as friendly (e.g., smiling, nodding, and relaxed tone) appeared in 98.02%. Close-up and medium-close shots were common, and all videos used vertical framing. These features reduce the status distance between a corporate leader and ordinary users, turning the entrepreneur from an organizational authority on a launch-event stage into an everyday figure on the screen.

Product props are crucial for this conversion. Xiaomi phones, the SU7 automobile, factory equipment, and digital devices are not only corporate information but also everyday objects embedded in the entrepreneur’s walking, testing, explaining, and responding. Users discuss products and Lei Jun and joke about Lei Jun while keeping the Xiaomi brand visible. Entrepreneurial identity capital, product functionality, and platformized everyday narratives jointly produce a life-like organizational persona.

### 4.2. Comment-Section Rituals Around Personhood, Products, and Fan/Consumer Identity

High-frequency words in the comment corpus concentrate on terms such as Xiaomi, Mr. Lei, phone, Lei Jun, car, SU7, Mi fans, customer service, product, price, and battery. These words simultaneously indicate the entrepreneur, Xiaomi products, and user needs. Therefore, the comment section does not merely provide feedback on video content. It becomes an interactional field in which entrepreneurial personhood, product issues, and fan/consumer identity are intertwined.

Thematic analysis identifies six major interaction themes: product requests, familiar address, emotional identification, memetic interaction, consumption mobilization, and brand defense. These themes correspond to a shared focus of attention, virtual co-presence, emotional resonance, group symbols, consumption imagination, and boundary maintenance. They can also be interpreted as differentiated forms of fan/consumer labor. Product requests perform informational labor by converting individual questions into a visible agenda for the entrepreneur, firm, and other users. Familiar address performs relational labor by reproducing an atmosphere of accessibility. Emotional identification performs affective and narrative labor by providing a shared moral meaning to the entrepreneurial experience. Memetic interaction performs cultural labor by generating reusable insider codes. Consumption mobilization performs promotional and projective labor by circulating scripts for saving, ordering, waiting, and future use. Brand defense performs boundary labor by defining legitimate criticism, defending product value, and distinguishing between supporters and perceived outsiders.

Product requests transform commercial issues into shared objects of attention. Beyond direct questions such as when an SU7 will be delivered, users recurrently produce paraphrasable requests such as “Could long-waiting owners receive a clearer delivery timetable?” and “Can the next version improve battery range and after-sales service?” These comments do more than express private needs. They keep delivery, price, configuration, camera performance, and service quality visible in the public feedback arena. By contrast, familiar address creates parasocial relationships. Although “Mr. Lei” remains common, users also employ more intimate and joking forms such as “Jun’er,” “Uncle Lei,” and “Brother Lei,” as well as expressions that can be paraphrased as “Brother Lei, remember to rest after the launch” or “Uncle Lei, please come to the comments and answer us.” Such address does not indicate real intimacy but performs everyday familiarity and makes organizational authority appear directly addressable.

Emotional identification transforms the entrepreneur’s experience into a group symbol. Comments about Lei Jun’s education, entrepreneurship, product launches, and Xiaomi’s development recurrently frame his story through paraphrasing such as “His persistence makes ordinary people feel that long-term effort matters” and “Following Xiaomi’s growth feels like witnessing an entrepreneur keep his promise.” Memetic interaction then condenses this shared history into insider language. “Are you OK,” joking wishes that Lei Jun earns “one million a year,” and suggestions that he could “leave Xiaomi and work independently” are repeatedly reproduced, including paraphrasable comments such as “The classic ‘Are you OK’ line is back again” and “With this workload, he should finally be paid one million a year.” Repetition is productive because it keeps the persona recognizable, lowers the threshold for participation, and provides users with a culturally shared way of displaying community membership (Table 6).

Consumption mobilization translates affection and product attention into public projections of future consumption. Comments include recurring patterns that can be paraphrased as “I have started saving for the SU7” and “After watching this, I want to place an order and wait for delivery.” Such statements are coded as consumption imagination because they narrate possible future acquisition or use and not because they verify payment or ownership. Brand defense creates a soft community boundary around the entrepreneur and product. Recurrent arguments can be paraphrased as “Compare the configuration before saying it is overpriced” and “Criticism is fair, but calling the product unserious ignores the engineering effort.” These comments reinterpret price, delivery, quality, and competitor comparisons through cost performance, technological progress, and the CEO’s perceived seriousness. Therefore, they contribute defensive labor while revealing the risk that product criticism may be moralized as disloyalty.

These findings show that the comment-section ritual is not one-way fan worship but a compound field in which users simultaneously act as fans, consumers, information providers, cultural producers, informal promoters, critics, and brand-community members. Fan labor is central because the persona is sustained not only by what the CEO posts but also by how users address him, repeat memes, formulate product agendas, circulate inspirational narratives, imagine purchases, and police community boundaries. Therefore, the platformized commercial persona is a distributed accomplishment of executive self-presentation, platform organization, audience discourse, and product-centered interaction. Furthermore, the term labor describes productive contribution rather than homogeneous intention. Some comments support the brand, some demand accountability, while others combine affection with criticism.

### 4.3. Emotional Intensity Across Interaction Themes

Emotional intensity analysis further shows that different themes play various roles in the interaction ritual. Although product requests are the largest category, they display relatively lower positive-emotion and high-intensity-expression ratios and mainly function as issue-raising and feedback entry points. Emotional identification, consumption mobilization, and brand defense show higher observable affective intensity. Emotional identification condenses shared admiration and moral evaluation. Consumption mobilization links affect public projections of saving, ordering, and waiting. Brand defense combines affective arguments with boundary maintenance. The component-wise results and composite pattern converge in suggesting that these themes more readily transform dispersed comments into shared affect, purchase-oriented imagination, and brand-community boundaries. This analysis does not assume that higher textual intensity is inherently more effective or authentic.

These results clarify the boundaries of this study’s claims. This study does not argue that comments directly produce purchase behavior, nor does it treat the emotional intensity index as a measure of stable attitude, persuasion, or psychological arousal. It shows that different forms of comment participation contain varying levels and configurations of observable affective expression, and that some themes are more closely associated with publicly articulated consumption imagination than others. Therefore, the interpretation rests on the convergence between dictionary-based signals, manual thematic validation, and interactional function of the comments (Table 7).

### 4.4. Integrating the Mechanism

**Figure 1:**
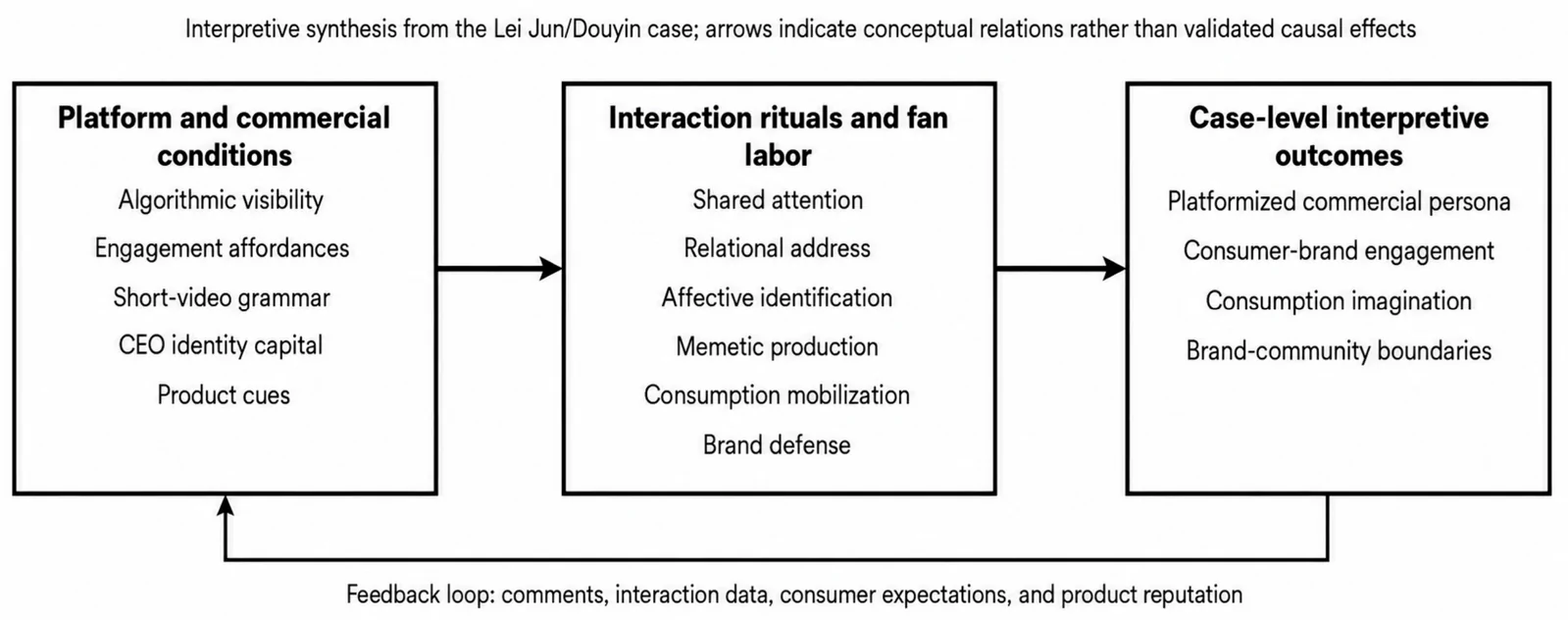
Case-derived conceptual framework of a platformized commercial persona in social commerce.

Figure 1 summarizes the case-derived conceptual framework of a platformized commercial persona in social commerce. It comprises three layers: platform and commercial conditions, interaction rituals and fan labor, and case-level interpretive outcomes. The arrows represent analytically reconstructed relationships supported by the content patterns and comment themes in this case. They do not constitute a statistically validated explanatory model, temporal sequence observed for every user, or evidence of causal effects. Platform affordances and commercial logic make interactions visible and repeatable. Audience labor organizes shared attention, relational closeness, cultural symbols, consumption projections, and boundaries. These processes may stabilize a platformized commercial persona, consumer–brand engagement, consumption imagination, and brand-community boundaries. The feedback loop indicates that comments, interaction data, consumer expectations, and product reputation can recursively shape subsequent content and interpretations. However, comparative and longitudinal studies are required to validate this framework beyond this case.

## 5. Discussion

### 5.1. Theoretical Contributions

The first contribution of this study is specifying the platformized commercial persona as an analytically bounded concept rather than as a general synonym for personal branding or CEO visibility. The concept identifies a public entrepreneurial identity with four linked properties: organizational authority, platform-shaped approachability, collective co-production, and commercial projection. Personal branding explains strategic self-positioning, CEO celebrity explains concentrated attention, influencer marketing explains branded persuasion, parasocial interaction explains relational closeness, and consumer–brand engagement explains audience investment. The platformized commercial persona explains how these elements are assembled around a CEO who directly embodies product responsibility and corporate reputation while being continuously reformatted by algorithmic visibility, short-video aesthetics, engagement affordances, and audience discourse. Therefore, the novelty of this study lies less in claiming that CEOs can be celebrities than in identifying the platformized interface through which executive authority is converted into addressability, product discussions, and commerce-oriented participation.

The second contribution is to make fan labor central to explaining CEO influencerization. Fan-labor research emphasizes that audiences not only consume but also interpret, circulate, promote, archive, and create value around cultural objects (Stanfill & Condis, 2014; Terranova, 2000). This study extends this insight to social commerce by identifying six observable forms of distributed labor: informational labor through product requests, relational labor through familiar address, affective and narrative labor through identification, cultural labor through memes, promotional and projective labor through consumption mobilization, and boundary labor through brand defense. This reframing clarifies why the persona cannot be attributed to executive self-presentation alone. It is maintained through repeated audience contributions that generate feedback agendas, recognizable symbols, emotional meanings, possible consumption scripts, and community boundaries. Furthermore, this analysis avoids the assumption that all participation is celebratory or willingly promotional. Demands, complaints, irony, and critical defense can also be productive components of persona construction.

The third contribution is extending interaction ritual chain theory to digital commerce while refining the meaning of consumption imagination. Shared attention and emotional energy are not spontaneous properties of fan communities. They are organized by recommendation systems, interface affordances, short-video grammar, product topics, and quantifiable engagement signals. CEO-centered rituals convert launch attention into repeated address, memes, affective identification, product requests, brand defense, and consumption-oriented projection. Consumption imagination names the publicly visible stage at which users narrate saving, ordering, waiting, owning, using, or recommending. This complements private consumption imagery research (Petrova & Cialdini, 2005; Phillips et al., 1995; Schlosser, 2003) by showing how future-oriented product projection can be socially circulated and collectively reinforced in a comment environment. Nevertheless, it remains analytically separate from purchase intention and actual conversion.

The fourth contribution is connecting CEO communication with social-commerce consumer engagement, without collapsing communication into sales effects. Research shows that personalization and conversational CEO communication can increase accessibility and relationship value (Men & Tsai, 2016; Yue et al., 2019; Denner & Schneider, 2023; Lee et al., 2026), whereas influencer research demonstrates the importance of credibility, authenticity, parasocial interaction, and message value (Hudders et al., 2021; Leung et al., 2022; Lou & Yuan, 2019; Sokolova & Kefi, 2020). This study supports these insights while adding a different structural condition in which the CEO is simultaneously an organizational leader, symbolic product guarantor, platform performer, and object of parasocial familiarity. Identity capital supplies credibility, everyday short-video narrative supplies approachability, fan labor supplies meaning and circulation, product discussion supplies a feedback interface, and consumption imagination connects affect to possible future use. Therefore, the case complements rather than contradicts prior work, while showing that executive visibility becomes commercially meaningful through a multi-actor, platform-mediated process.

### 5.2. Practical Implications for Social-Commerce and Brand Managers

For social-commerce and digital marketing practice, the key issue is not simply whether a CEO appears on camera. The more important question is whether a brand can build a stable, credible, and sustainable interaction persona that links executive visibility to product trust and consumer participation. Lei Jun’s account suggests that effective CEO short-video communication depends on the fit between work footage, product narrative, approachable presentation, and comment-section participation. Brands should avoid turning CEO accounts into advertising display windows. Instead, they should be used as interactional interfaces through which product information, consumer concerns, and brand values can be connected repeatedly.

However, CEO influencerization entails risks for social commerce. Excessive entertainment may weaken perceived professional authority; fan-style interaction may simplify complex product problems into emotional alignment; and algorithmic preference for controversy or high-arousal engagement may push executive communication toward exaggeration or personality dependence. When product quality, delivery, or after-sales problems arise, highly personalized communication can concentrate public pressure on the CEO. The visibility created by fan labor should not be treated as a substitute for formal customer service or freely exploitable promotional output. Brands should acknowledge recurrent user contributions, provide transparent routes for product feedback, avoid mobilizing defensive fandom against legitimate criticism, and balance emotional participation with product accountability and risk-management mechanisms.

### 5.3. Limitations and Future Research

This study has several limitations. First, it uses a single-case design. Lei Jun is a highly visible technology entrepreneur, and while this case is analytically revealing, it is not representative of all CEO accounts, brands, industries, or social-commerce platforms. Therefore, Figure 1 presents a case-derived conceptual framework, rather than an empirically validated explanatory model. Comparative research should examine entrepreneurs across industries, ownership types, product categories, cultural settings, and platform architectures, and test whether the proposed dimensions and relationships extend beyond this case. Second, this study mainly uses public short-video and comment data. Although BERTopic-assisted topic location, manual validation, intercoder reliability testing, and emotional intensity analysis improve transparency, they cannot directly reveal users’ motives, self-identification as fans, perceived labor, trust formation, or decision journeys. The term “fan labor” is inferred from the productive interactional functions of visible comments, rather than from interviews about participants’ intentions. Future studies should combine text analysis with surveys, interviews, digital ethnography, clickstream data, experiments, or platform-level exposure data.

Third, consumption imagination is identified through public linguistic projections of saving, ordering, waiting, use, and recommendation. These expressions are not equivalent to individual purchase intentions and cannot verify actual payment, ownership, or long-term loyalty. Fourth, the emotional intensity indicator captures observable textual expression rather than psychological states. The platform-specific dictionary was derived through contextual manual validation, and the four standardized components were equally weighted because no validated basis supported differential weights. Humor, irony, memes, and formulaic repetition may produce high scores without indicating stable attitudes. Future research should validate these indicators using human ratings and user-level measures. Fifth, the supplementary regional association check cannot identify causal relationships. Population, income, automobile consumption base, infrastructure, store distribution, delivery capacity, platform exposure, and regional preferences may influence comments and sales. Longer time-series, city-level data, event-study designs, fixed-effects models, and quasi-experimental settings are required to examine communication, product attention, and conversion.

## 6. Conclusions

Regarding RQ1, CEO short videos are characterized by a combination of organizational and everyday interaction resources. Work footage and scenes of daily life dominate the account, while casual clothing, friendly expression, close visual framing, and product props translate executive authority into an approachable mediated presence. These features do not remove organizational authority. Instead, they reformat it into a public persona that is professional, familiar, and product-connected.

Regarding RQ2, comment-section interactions transform CEO personhood, product issues, and fan/consumer identity through six linked ritual practices and forms of labor. Product requests create shared agendas, familiar address performs relational closeness, emotional identification provides a collective moral meaning to entrepreneurial experience, memes produce group symbols, consumption mobilization circulates future-oriented consumption scripts, and brand defense negotiates community boundaries. Therefore, the platformized commercial persona is not authored by the CEO alone but co-produced through platform mechanisms, executive content, fan/consumer discourse, and product accountability.

Regarding RQ3, the interaction themes vary in terms of observable emotional intensity. Product requests are frequent but comparatively less affectively intense, whereas emotional identification, consumption mobilization, and brand defense display stronger positive and high-intensity textual signals. This variation helps explain how some commenting practices move beyond issue-raising to condense emotional energy, activate publicly expressed consumption imagination, and maintain brand-community boundaries. These findings do not establish that high-intensity comments cause purchases, but identify differentiated interactional functions in a visible comment environment.

Collectively, the findings support previous research showing that personalized CEO communication can enhance approachability and relational value and complement influencer-marketing research on credibility, authenticity, and parasocial interaction. They extend this literature by demonstrating that CEO influencerization is a multi-actor platform process in which organizational authority, algorithmic visibility, short videos, fan labor, and product-centered imagination are structurally coupled. This study did not formulate causal hypotheses, and its contribution is conceptual and interpretive, offering a bounded construct and case-derived framework for comparative research on entrepreneurial communication in global social-commerce environments.

## Figures and Tables

**Table 1:** Analytical boundaries of the platformized commercial persona.

Neighboring Concept	Primary Analytical Focus	Why It Is Insufficient Alone	Distinctive Focus of Platformized Commercial Persona
Personal branding	Strategic self-positioning and reputation management	Centers the focal actor’s intentional self-management	Includes algorithmic sorting, short videos, engagement affordances, and distributed comment labor in persona production
CEO celebrity	Accumulation of attention, status, and recognizability around executives	Does not necessarily explain how attention becomes product discussion or commerce-related participation	Tracks the translation of executive visibility into product feedback, brand participation, and consumption imagination
Influencer marketing	Creator credibility, branded persuasion, endorsement, and purchase effects	Usually treats the influencer as external to the focal firm or product responsibility	Examines a CEO who simultaneously embodies organizational authority, product responsibility, corporate reputation, and platform performance
Parasocial interaction	Perceived intimacy and one-sided relational closeness	Primarily explains a user–persona relationship	Adds platform mechanisms, fan-to-fan interaction, product issues, and collective boundary work
Consumer–brand engagement	Cognitive, emotional, and behavioral investment in brand relations	Primarily conceptualizes an engagement state or outcome	Treats the CEO persona as a platformized interface through which engagement is collectively organized and commercial meanings are produced

**Table 2:** Video content analysis coding dimensions.

Category	Subcategory	Coding Purpose
Narrative attributes	Main theme, temporal structure	Identifies whether the video centers on business updates, work footage, everyday life, life history, or other narrative forms.
Presentation status	Clothing, action, facial expression	Captures whether the entrepreneur is presented as formal, relaxed, friendly, or every day.
Scene setting	Location, actors, props	Records office, outdoor, factory, launch event, employees, peers, products, and other visible interaction cues.
Symbolic elements	Stickers, music, language, editing, shot size	Examines how short-video grammar enhances visibility, platform style, and interaction invitation.
Communication elements	Duration, frame, likes, comments, hashtags	Documents quantified interaction and distribution-related cues.

**Table 3:** Intercoder reliability for video content analysis.

Coding Dimension	Reliability Statistic	Interpretation
Main theme	Cohen’s kappa = 0.86	High agreement
Scene setting	Cohen’s kappa = 0.83	High agreement
Presentation status	Cohen’s kappa = 0.81	Acceptable-to-high agreement
Symbolic elements	Cohen’s kappa = 0.79	Acceptable agreement
Communication elements	Cohen’s kappa = 0.88	High agreement
Overall average	Cohen’s kappa = 0.83	Acceptable level for formal analysis

**Table 4:** Comment themes identified through BERTopic assistance and manual validation.

Theme	Keywords/Representative Cues	Share	Manual Validation	Theoretical Meaning
Product requests	SU7, delivery, price, after-sales, battery, configuration	31.6%	Reviewed product consultation, delivery waiting, and configuration suggestion comments.	Shared focus of attention
Familiar address	Mr. Lei, Jun’er, Uncle Lei, Brother Lei, take care	18.4%	Reviewed address forms, greetings, and quasi-familiar expressions.	Parasocial closeness
Emotional identification	Support, inspiring, ordinary people, seriousness	15.7%	Reviewed value identification and entrepreneurship narrative comments.	Emotional energy
Memetic interaction	Are you OK, one million a year, leave Xiaomi	14.2%	Reviewed memes, parody, and insider references.	Group symbols
Consumption mobilization	Place an order, rush SU7, wait for delivery, save money	11.3%	Reviewed purchase desire, collection, and potential consumption expressions.	Consumption imagination
Brand defense	Cost-performance, technology, serious product-making, do not smear	8.8%	Reviewed responses to criticism and brand-defense comments.	Boundary maintenance

**Table 5:** Construction and interpretive boundaries of the emotional intensity indicator.

Component	Operational Rule	Example Cues	Analytical Rationale	Interpretive Boundary
Positive affect	Positive-emotion word hits normalized by comment length	support; moved; inspiring	Captures observable positive valence.	Textual tendency only; not a psychological state.
Lexical intensification	Degree adverbs and high-intensity emotion words	very; truly; so strong	Captures lexical amplification of affect.	May include conventional or ironic usage; context was manually checked.
Punctuation/emoji reinforcement	Exclamation marks, repeated punctuation, and affective emojis identified by regular expressions	rush!!!; repeated question marks; laughing/crying emoji	Captures platform-style paralinguistic reinforcement.	Cannot independently determine valence or sincerity.
Action/boundary orientation	Action-oriented consumption terms or brand-defense/boundary terms	order; save money; wait for delivery; do not smear	Connects affective expression to consumption projection or boundary work.	Not direct purchase evidence or proof of stable loyalty.
Composite index	Equal-weight mean of the four standardized components	EI_c = [z(Pos_c) + z(Int_c) + z(PuncEmoji_c) + z(Orient_c)]/4	Makes components with different raw scales comparable across themes.	Exploratory auxiliary indicator; interpreted with component ratios and qualitative context.

**Table 6:** Comment themes, ritual mechanisms, and fan-labor contributions.

Theme	Illustrative Composite Paraphrases	Ritual Mechanism	Interaction Function	Fan-Labor Contribution
Product requests	“Could owners receive a clearer delivery timetable?” “Can the next version improve battery range and after-sales service?”	Shared focus	Turns product issues into durable public topics.	Informational and feedback labor
Familiar address	“Brother Lei, remember to rest after the launch.” “Uncle Lei, please answer us in the comments.”	Virtual co-presence	Reduces status distance and simulates direct conversation.	Relational labor
Emotional identification	“His persistence makes ordinary people believe long-term effort matters.” “Following Xiaomi’s growth feels like witnessing a promise being kept.”	Emotional resonance	Turns entrepreneurial experience into a shared moral symbol.	Affective and narrative labor
Memetic interaction	“The classic ‘Are you OK’ line is back.” “With this workload, he should finally earn one million a year.”	Group symbols	Creates insider language and lowers the threshold for participation.	Cultural and memetic labor
Consumption mobilization	“I have started saving for the SU7.” “After watching this, I want to place an order and wait for delivery.”	Consumption imagination	Projects possible future acquisition and use.	Promotional and projective labor
Brand defense	“Compare the configuration before calling it overpriced.” “Criticism is fair, but the engineering effort should not be dismissed.”	Boundary maintenance	Reframes criticism and negotiates legitimate brand-community boundaries.	Defensive and boundary labor

Note: Table 6 presents comment-section themes, additional paraphrased examples, ritual mechanisms, and fan-labor contributions. The examples provided are composite paraphrases of recurrent coded expressions rather than verbatim quotations.

**Table 7:** Emotional intensity indicators across comment themes.

Theme	Positive-Emotion Ratio	High-Intensity-Expression Ratio	Main High-Intensity Cues	Ritual Function
Product requests	42.8%	18.6%	Price, delivery, and configuration questions with relatively limited strong affect	Raises shared issues
Familiar address	71.2%	35.4%	Familiar address, greetings, joking, emojis	Creates parasocial closeness
Emotional identification	78.9%	46.1%	Support, moved, inspiring, ordinary-person narratives	Condenses emotional energy
Memetic interaction	69.5%	40.7%	Repeated memes, exaggeration, repeated punctuation	Forms group symbols
Consumption mobilization	73.6%	43.8%	Rush, order, wait for delivery, save money	Activates consumption imagination
Brand defense	66.9%	38.5%	Responding to criticism, defending the brand, emotional argumentation	Maintains community boundaries

## Data Availability

The raw comment dataset cannot be publicly shared due to platform terms of service and user privacy considerations. An anonymized coding scheme and aggregated analytical materials are available from the author upon reasonable request.

## Appendix A. Supplementary Contextual Check of Regional Platform Attention

This supplementary contextual check is reported only as background information. It is not used as a core result and does not support causal claims regarding sales conversion.

Before 28 March 2024, the dataset contained 68,775 comments. After removing comments without visible provincial IP information, 39,261 remained for provincial aggregation. Guangdong, Zhejiang, Jiangsu, Shandong, and Henan had relatively high comment counts. A third-party SU7 sales ranking for April 2024 indicated that Guangdong, Beijing, Zhejiang, Jiangsu, and Shanghai were among the leading regions. The sales list is used only as an auxiliary market-attention indicator because it is a third-party industry ranking rather than Xiaomi’s audited sales disclosure.

The descriptive check shows positive co-movement between provincial comment IP counts and provincial SU7 sales (Pearson’s r = 0.805, *p* < 0.001). Spearman’s rank correlation remains positive (rho = 0.762, *p* < 0.001). After log(x + 1) transformation of both variables, the correlation coefficient was r = 0.731 (*p* < 0.001). The leave-one-out procedure produced Pearson’s correlations, ranging from 0.716 to 0.842, all of which were significant at *p* < 0.001. These results indicate that visible interaction intensity and market attention moved in the same direction within this limited third-party dataset. However, they do not demonstrate that short-video comments caused sales.

**Table A1:** Robustness description of the supplementary contextual check of regional platform attention.

Test	Statistic/Model	Coefficient or Range	Significance	Interpretation
Pearson correlation	Provincial comment IP counts and SU7 provincial sales	r = 0.805	*p* < 0.001	Positive descriptive co-movement at the provincial level.
Spearman rank correlation	Ranked comment counts and regional sales	rho = 0.762	*p* < 0.001	Co-movement remains positive under rank order.
Log-transformed correlation	log (comment IP count + 1) and log(sales + 1)	r = 0.731	*p* < 0.001	Positive after reducing the leverage of high-value regions.
Leave-one-out check	Remove one province at a time and recalculate Pearson correlation	r = 0.716–0.842	all *p* < 0.001	The pattern does not depend on a single region.
Interpretive boundary	Supplementary contextual check, not causal identification	—	—	Third-party sales data, small provincial samples, and potential confounders limit the interpretation.
